# OLFACTION AND COGNITIVE FUNCTION IN NONDEMENTED OLDER ADULTS: FINDINGS FROM HEALTH ABC

**DOI:** 10.1093/geroni/igae098.3321

**Published:** 2024-12-31

**Authors:** Yaqun Yuan, Keran Chamberlin, Chenxi Li, Zhehui Luo, Anna Kucharska-Newton, Srishti Shrestha, Eleanor Simonsick, Honglei Chen

**Affiliations:** Michigan State University East Lansing, East Lansing, Michigan, United States; Michigan State University, United States; Michigan State University East Lansing, East Lansing, Michigan, United States; Michigan State University East Lansing, East Lansing, Michigan, United States; University of North Carolina at Chapel Hill, Chapel Hill, North Carolina, United States; University of Mississippi Medical Center, Jackson, Mississippi, United States; National Institute on Aging, Baltimore City 0400 Baltimore City, Maryland, United States; Michigan State University, Michigan State University, Michigan, United States

## Abstract

We examined olfaction in relation to longitudinal change in cognitive function among 1987 community-dwelling participants of the Health, Aging and Body Composition study (aged 71-82 years, 52.8% women, and 33.1% Black race), free of dementia and Parkinson’s disease. Participants completed the Brief Smell Identification Test at the year 3 clinic visit in 1999-2000. Olfaction was defined as good (test score 11-12), moderate (9-10), hyposmia (7-8), or anosmia (0-6). Cognitive function was assessed using the Modified Mini-Mental State examination (3MS) up to 5 times at clinic visits in year 3, 5, 8, 10 and 11. We conducted multivariable joint model analyses to account for potential bias due to death and loss to follow-up, in addition to potential confounders. Olfaction status was inversely associated with 3MS test score in both cross-sectional and longitudinal analyses over 8 years of follow-up. At baseline, compared with participants with good olfaction, the 3MS test score was 0.80 (95%CI, 0.21-1.38) points lower for those with moderate olfaction, 1.82 (95%CI, 1.08-2.57) points lower for those with hyposmia, and 1.87 (95%CI, 1.00-2.75) points lower for those with anosmia. Longitudinally, the annual decline was 0.09 (95%CI, 0.01-0.17) faster for those with moderate olfaction, 0.22 (95%CI, 0.11-0.32) faster for those with hyposmia, and 0.30 (95%CI, 0.17-0.43) for those with anosmia. Similar associations were observed across subgroups defined by sex, race, self-reported general health status, and APOE-ε4 genotype. Our study provides important evidence that poor olfaction is associated with a faster decline in cognitive function in non-demented older adults.

